# Performance of AI to exclude normal chest radiographs to reduce radiologists’ workload

**DOI:** 10.1007/s00330-024-10794-5

**Published:** 2024-05-17

**Authors:** Steven Schalekamp, Kicky van Leeuwen, Erdi Calli, Keelin Murphy, Matthieu Rutten, Bram Geurts, Liesbeth Peters-Bax, Bram van Ginneken, Mathias Prokop

**Affiliations:** 1 Department of Imaging, Radboudumc, Nijmegen, The Netherlands; 2 Department of Radiology, Jeroen Bosch Ziekenhuis, ’s Hertogenbosch, The Netherlands

**Keywords:** Radiography (thoracic), Respiratory tract diseases, Diagnosis (computer-assisted), Artificial intelligence, Workload

## Abstract

**Introduction:**

This study investigates the performance of a commercially available artificial intelligence (AI) system to identify normal chest radiographs and its potential to reduce radiologist workload.

**Methods:**

Retrospective analysis included consecutive chest radiographs from two medical centers between Oct 1, 2016 and Oct 14, 2016. Exclusions comprised follow-up exams within the inclusion period, bedside radiographs, incomplete images, imported radiographs, and pediatric radiographs. Three chest radiologists categorized findings into normal, clinically irrelevant, clinically relevant, urgent, and critical. A commercial AI system processed all radiographs, scoring 10 chest abnormalities on a 0–100 confidence scale. AI system performance was evaluated using the area under the ROC curve (AUC), assessing the detection of normal radiographs. Sensitivity was calculated for the default and a conservative operating point. the detection of negative predictive value (NPV) for urgent and critical findings, as well as the potential workload reduction, was calculated.

**Results:**

A total of 2603 radiographs were acquired in 2141 unique patients. Post-exclusion, 1670 radiographs were analyzed. Categories included 479 normal, 332 clinically irrelevant, 339 clinically relevant, 501 urgent, and 19 critical findings. The AI system achieved an AUC of 0.92. Sensitivity for normal radiographs was 92% at default and 53% at the conservative operating point. At the conservative operating point, NPV was 98% for urgent and critical findings, and could result in a 15% workload reduction.

**Conclusion:**

A commercially available AI system effectively identifies normal chest radiographs and holds the potential to lessen radiologists’ workload by omitting half of the normal exams from reporting.

**Clinical relevance statement:**

The AI system is able to detect half of all normal chest radiographs at a clinically acceptable operating point, thereby potentially reducing the workload for the radiologists by 15%.

**Key Points:**

- *The AI system reached an AUC of 0.92 for the detection of normal chest radiographs*.
- *Fifty-three percent of normal chest radiographs were identified with a NPV of 98% for urgent findings. AI can reduce the workload of chest radiography reporting by 15%*.

## Introduction

Chest radiography is the most commonly used radiological exam to identify or exclude various thoracic diseases [1]. This is one of the reasons why there are now several artificial intelligence (AI) systems for chest radiography commercially available [2]. Most AI systems are developed to detect specific abnormalities in chest radiographs, such as: pneumonia, lung nodules, tuberculosis, or pneumothorax. Other products have been developed to detect the most common chest abnormalities or try to evaluate the whole spectrum of thoracic diseases and deliver a report to the radiologist [2–5].

Aside from the potential of AI systems to enhance the quality of chest radiography interpretation [6–10], they could also aim to reduce the reporting time by radiologists [6, 11]. Therefore, several studies focused on triaging chest radiographs, prioritizing urgent findings in the radiologist’s worklist, and ensuring that they are reported first [12–14]. Another effective strategy for saving time is by reliably excluding the presence of any relevant chest radiograph abnormality, thereby eliminating the need for further evaluation by a radiologist. From both a radiological and societal point-of-view, it would be interesting to eliminate (redundant) reporting of these normal images, especially in an era of increasing workload for radiologists, shortage of physicians, and societal pressure to keep healthcare budgets stable [15–20].

Previous studies have estimated that around 28–36% of normal chest radiographs could be identified using an AI system, with high confidence. This could lead to a reduction of 8–17% of the total number of chest radiographs that need to be reported by the radiologist [21–23]. However, data on the potential of AI to eliminate radiological exams from reporting by a radiologist are scarce, and very little is known about the generalizability of such solutions. Further, the performance is dependent on the operating point of the AI system, which has not been investigated in previous studies.

The objective of this study is to investigate the potential of a commercially available AI product to identify normal chest radiographs in a consecutive clinical series of radiographs from two different hospitals. We evaluated different operating points and estimated the percentage of normal chest radiographs that could be safely eliminated from the usual clinical workflow.

## Methods

### Ethics approval statement

Ethical approval will be waived as this is retrospective use of clinically acquired data (local institution review board approval number 2023-16333). Radiographs and reports were anonymized before analysis. This manuscript was prepared according to the Standards for Reporting of Diagnostic Accuracy Studies checklist [24].

### Study population

All consecutive chest radiographs and their radiological reports between 1st October 2016 and 14th October 2016 were retrospectively collected from an academic hospital (Radboudumc, Nijmegen, The Netherlands) and a large community hospital (Jeroen Bosch Hospital, ‘s Hertogenbosch, The Netherlands). The study population included inpatient, outpatient, and emergency chest radiographs. Only the first initial exam in our inclusion period for each patient was included to prevent bias of multiple exams from the same patient. Patients could have had prior exams before the inclusion period. Bedside radiographs, pediatric and incomplete visualized chests, as well as the radiographs imported from other hospitals were excluded, based on DICOM tags or visual inspection.

### Reference standard

The reference standard was set by three chest radiologists (S.S., 9 years of experience; B.G. 12 years of experience; L.P.B. 20 years of experience) who independently reviewed the exams with both the PA and lateral radiograph (when available). In this setup there was no access to AI results, prior and follow-up CXR and/or chest CT exams. However, the radiologists had access to the original report, including medical history and indication. No AI software has been used at the time of original reporting of the chest radiographs included in this study.

The radiologists categorized the chest radiographs into the following clinical categories: normal, clinically irrelevant findings (e.g., medical devices), clinically relevant findings (e.g., cardiomegaly), urgent findings (e.g., air space consolidation), and critical findings (e.g., pneumothorax). The majority vote was used to assign the clinical categories to the chest radiographs. When no majority was reached, the mean category (where normal represents a score of 1, and critical findings a score of 5) of the three raters was used. All findings and their corresponding clinical category can be found in Table 1.

**Table 1:** Classification of chest radiography findings according to the reference standard

Normal	Non-clinically relevant findings	Clinically relevant findings	Urgent findings	Critical findings
No abnormalities	Medical devices	Hiatus hernia	Atelectasis	Pneumomediastinum
	Sternal cerclages	Cardiomegaly	Air space consolidation	Pneumothorax
	Aortic calcification	Elevated diaphragm	Bone lesions/rib fractures	Subcutaneous emphysema
	Vertebral spondylosis	Old rib fractures	Interstitial abnormalities	Pneumoperitoneum
	Mild bronchopathy	Pleural calcification	Hilar enlargement	Malpositioned line/medical device
		Plate atelectasis	Mass or nodule	
		Bronchiectasis	Pleural effusion	
		Pectus excavatum		
		Vertebral collapse		

### Analysis by AI

All included images were processed by Lunit INSIGHT CXR3 (version 3.1.4.4). Lunit INSIGHT CXR3 is a CE-certified AI product (Medical Device Regulation class IIa) that evaluates posteroanterior and anteroposterior chest radiographs to detect abnormalities. The AI system does not consider previous radiographs and it does not analyze lateral radiographs. The AI system provides an abnormality score for ten different thoracic abnormalities (atelectasis, consolidation, fibrosis, calcification, nodules, cardiomegaly, mediastinal widening, pleural effusion, pneumothorax, and pneumoperitoneum). The abnormality scores were in the range of 0–100. The AI system is not designed to detect normal chest radiographs. Therefore we calculated a ‘normality’. This was subsequently defined as a 100-maximum score of any abnormality. If the normality score was above the defined threshold, the case was considered normal.

### Statistical analysis

The area under the ROC curve (AUC) was used to assess the performance of the AI system in detecting normal chest radiographs. We evaluated the performance of the AI system at two different operating points: an operating point that corresponds with a specificity of 95% on the AUC, hereafter called a conservative operating point, and the default recommended operating point of the AI system. The default recommended operating point is designed for the detection of abnormal findings, and not designed to detect normal chest radiographs. At these two operating points, the sensitivity for detecting of normal chest radiographs, and the negative predictive value (NPV) for the urgent and critical findings was calculated. For both operating points the potential workload reduction was calculated by dividing the number of chest radiographs classified as normal with the total number of chest radiographs from our inclusion period, excluding follow-up exams and radiographs imported from other hospitals. False negative urgent/critical findings were analyzed at the conservative operating point. The statistical analysis was performed using SPSS (IBM statistics, version 29). *p* Values < 0.05 were considered significant.

## Results

### Patient population

**Fig. 1:**
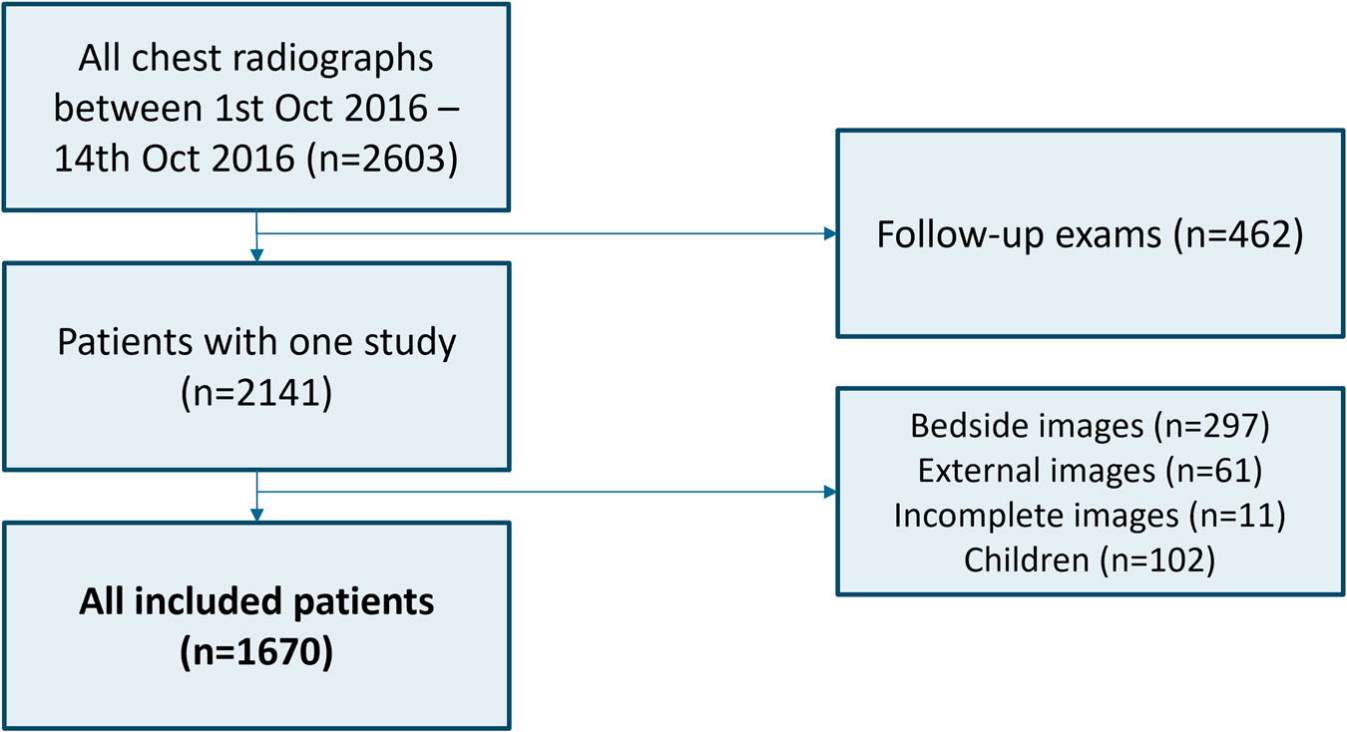
Exclusion flowchart of study participants

A total of 2603 radiographs were acquired in 2141 unique patients. After exclusion of follow-up exams (*n* = 462), bedside radiographs (*n* = 297), imported radiographs (*n* = 61), incomplete radiographs (*n* = 11), and pediatric radiographs (*n* = 102), 1670 radiographs (patient median age 61 (IQR 48–71), M:F 868:802) were included for analysis (Fig. 1). For 1583 of 1670 cases (95%) a lateral chest radiograph was available. Among the included cases, 479 were normal, 332 had clinically irrelevant findings, 339 had clinically relevant findings, 501 had urgent findings, and 19 contained critical findings. The distribution of findings across the hospital is displayed in Table 2.

**Table 2:** Distribution of findings on chest radiographs acquired in both participating hospitals

Findings	Hospital 1, (*n* = 680)	Hospital 2, (*n* = 990)	Total, (*n* = 1670)
Normal	203 (29.9%)	276 (27.9%)	479 (28.7%)
Non clinically relevant	147 (21.6%)	185 (18.7%)	332 (19.9%)
Clinically relevant	119 (17.5%)	220 (22.2%)	339 (20.3%)
Urgent	201 (29.6%)	300 (30.3%)	501 (30.0%)
Critical	10 (1.5%)	9 (0.9%)	19 (1.1%)

### Performance of the AI system for the detection of normal chest radiographs

**Fig. 2:**
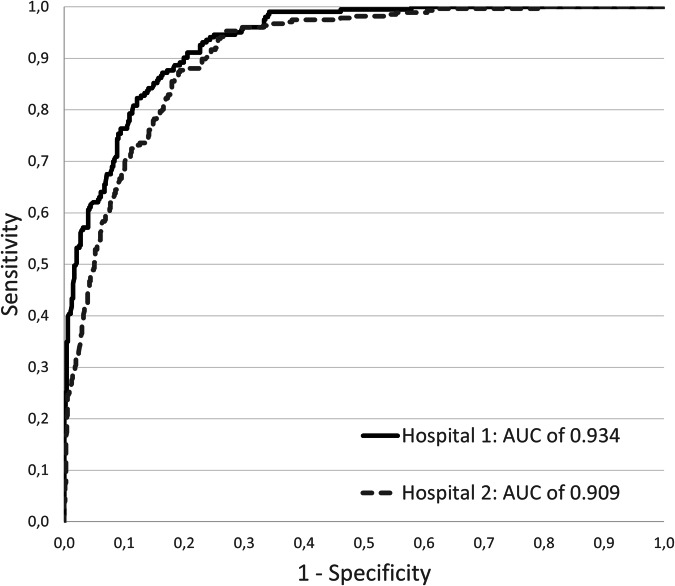
Receiver operating characteristics (ROC) curve for the detection of normal radiographs by the AI system on a consecutive series of chest radiographs from two Dutch medical centers (*n* = 1670). AUC, area under the curve

The AUC of the AI system was 0.918 (95% CI 0.905–0.931) for the detection of normal cases (Fig. 2). No significant difference in AI performance was found between the hospitals (AUC = 0.934 (95% CI 0.916–0.951) for hospital 1 vs AUC = 0.909 (95% CI 0.891–0.928) for hospital 2; *p* = 0.06). At our conservative operating point, the AI system marked 314 (18.8%) out of 1670 radiographs as normal, 254 of these were normal according to our reference standard. This corresponds with a sensitivity of 53% (254/479). If these radiographs were eliminated from the usual clinical workflow the total workload reduction (including bedside, pediatric, and incomplete chest radiographs) would amount to 314 of 2080 (15.1%) radiographs.

**Fig. 3:**
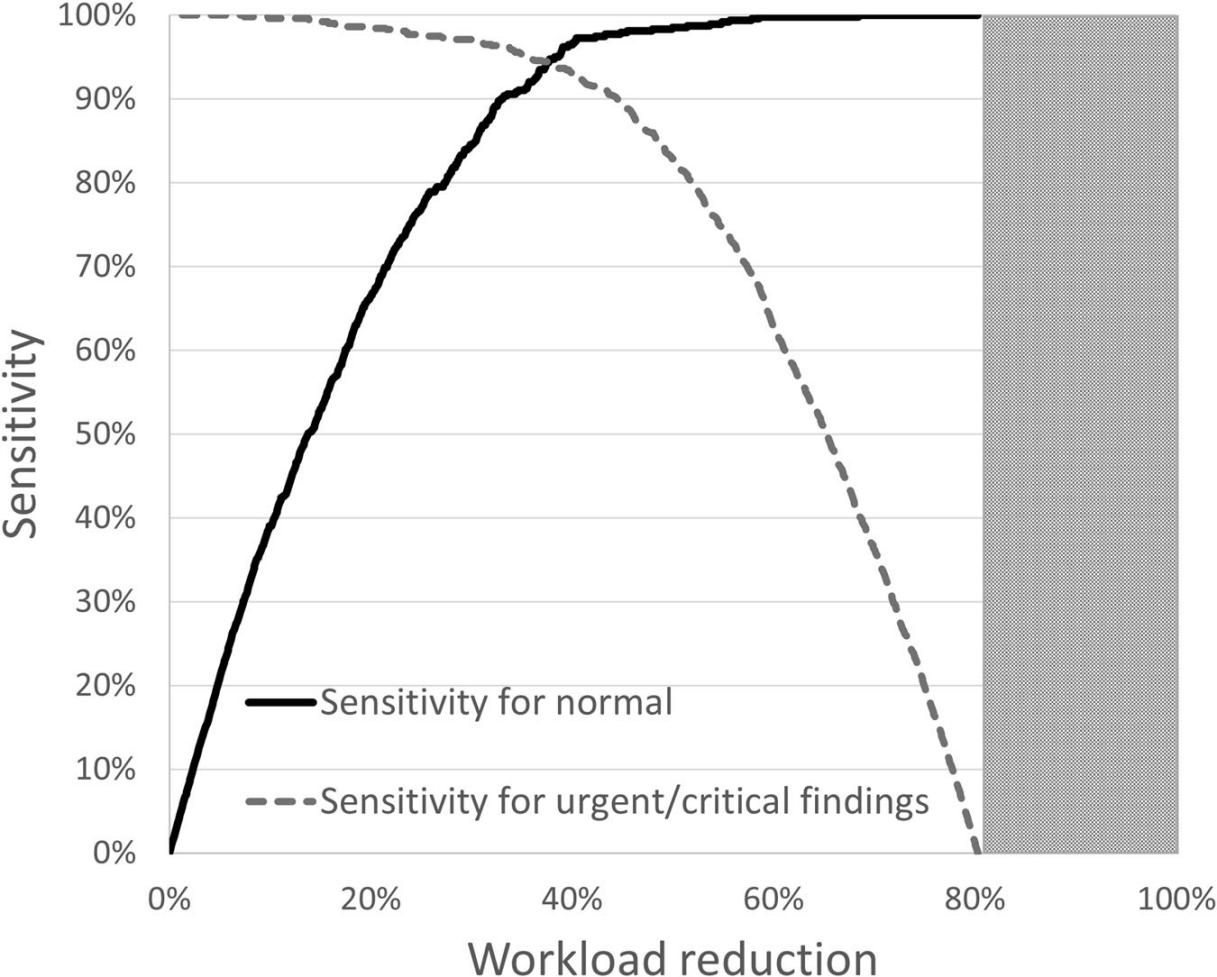
Potential workload reduction compared with the sensitivity of the AI system for normal radiographs (black line) and the sensitivity for urgent/critical findings. The graph shows the tradeoff of increased workload reduction at the cost less sensitivity for urgent/critical findings. The maximum workload reduction is 1670/2080 (analyzed radiographs/analyzed radiographs + excluded radiographs) = 80.3%. Higher workload reduction (gray area) could not be achieved in this setup

At the default operating point (vendor-recommended operating point), the AI system detected 442 of the 479 normal chest radiographs (92.3%). Among the 1670 included radiographs, AI identified 753 chest radiographs as normal (45.1%). This would result in a total workload reduction of 753 of 2080 (36.2%) chest radiographs (Fig. 3).

### Abnormality detection and error analysis

**Fig. 4:**
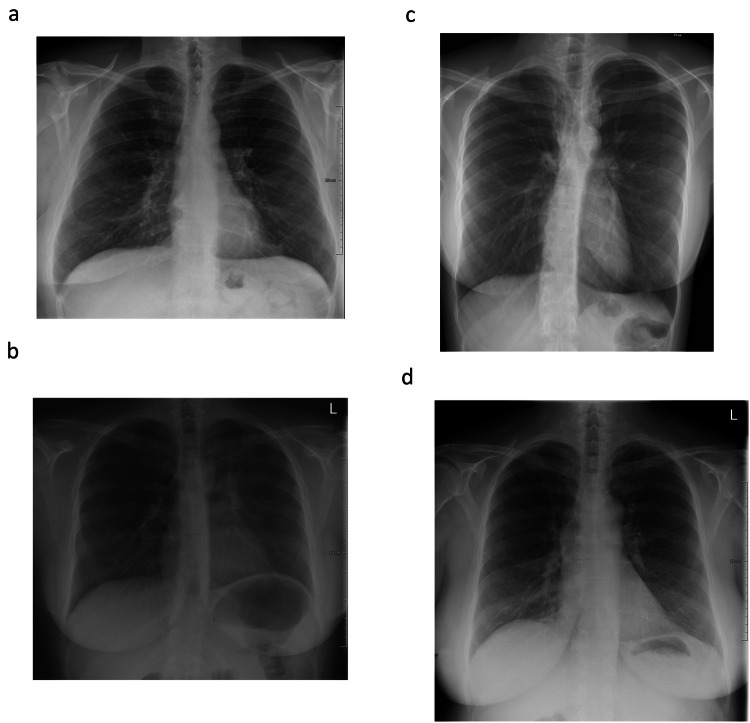
The four cases with urgent findings missed by the AI system at the conservative operating point. **a** From earlier CT known stable nodule in the right lower quadrant, with characteristics of a subpleural lymph node on CT. Classified as an urgent finding by two of the three radiologists. **b** CT confirmed left hilar mass. Classified as an urgent finding by all three radiologists. **c** Suspicion of minor post-tuberculosis fibrotic changes in the upper right lung. Classified as an urgent finding by two of the three radiologists. **d** In the original report description of small right paracardial infiltrate. Classified as an urgent finding by two of the three radiologists

At our conservative operating point, AI misclassified 60 radiographs as normal while the reference standard reported abnormalities. Of these, 45 radiographs contained clinically irrelevant findings, 11 radiographs had clinically relevant findings, and 4 showed urgent findings (Table 3). None of the critical cases were classified as normal by the AI system. At the conservative operating point 287 of 332 (86.4%) clinically irrelevant findings, 328 of 339 (96.8%) clinically relevant findings, 497 of 501 (99.2%) urgent findings, and all 19 (100%) critical findings would not be classified as normal. This would correspond to an NPV of 81% for all abnormalities, 94% after the exclusion of clinically irrelevant findings, and 98% after the exclusion of both clinically irrelevant and relevant findings (Table 4). The four misclassified urgent findings were a hilar mass (CT confirmed), small paracardial consolidation (not CT confirmed), a description of a small nodule in the RLL that was a subpleural nodule lymph node on previous and follow-up CT, and suspected minor post-tuberculosis changes (Fig. 4). At the default recommended operating point designed to find abnormalities rather than normal images, the AI erroneously classified 311 radiographs as normal. Out of these, 202 radiographs contained clinically irrelevant findings, 81 radiographs had clinically relevant findings and 28 showed urgent findings (Table 3). At this default operating point, the AI system remained sensitive to urgent findings 473/501 (94.4%) and did not misclassify any of the critical findings (Table 4).

**Table 3:** Distribution of findings present in the normal classified radiographs by the AI system at the conservative and the default operating point

Findings reference standard	AI normal classification at conservative operating point, (*n* = 314)	AI normal classification at the default recommended operating point, (*n* = 753)	Total, (*n* = 1670)
Normal	254	442	479
Clinically irrelevant	45	202	332
Clinically relevant	11	81	339
Urgent	4	28	501
Critical	0	0	19

*AI* artificial intelligence

**Table 4:** Sensitivity, specificity, NPV, and positive predictive value of the AI system for the detection of all abnormalities, or urgent and critical findings only

Accuracy for abnormal findings	Conservative operating point	Default operating point
All abnormal findings		
Sensitivity	95% (1131/1191)	74% (880/1191)
Specificity	53% (254/479)	92% (442/479)
NPV	81% (254/314)	59% (442/753)
PPV	83% (1131/1356)	96% (880/917)
Urgent/critical findings		
Sensitivity	99% (516/520)	95% (492/520)
Specificity	53% (254/479)	92% (442/479)
NPV	98% (254/258)	94% (442/470)
PPV	70% (516/741)	93% (492/529)

Values are displayed for both the conservative and the default operating point

*NPV* negative predictive value, *PPV* positive predictive value

## Discussion

This multicenter study shows that half of the normal chest radiographs can be identified by AI at a clinically acceptable threshold while remaining very sensitive to urgent and critical findings. This holds the potential to reduce the workload for radiologists by 15% for the reporting of chest radiographs.

Our results are in line with previous publications that showed a potential total workload reduction of 8–17%. However, the calculated workload reduction is dependent on several factors including the quality of the AI system, the chosen operating point of the AI system, the definition/ground truth of normal chest radiograph, the population, and which radiographs were considered when calculating the reduction of workload.

Our population may have differed from previous studies. In the study by Plesner et al [23], the population was slightly older (69 vs 61 years of age) but the percentage of normal radiographs was 28% which is comparable to our percentage of 29%. In the study by Keski et al [22] no population demographics were given and the percentage of normal chest radiographs was 46–48%. However, this study used radiographs from a primary healthcare setting, which may represent a healthier population than in our study. Nonetheless, AI systems seem to be robust to these population differences, considering the comparable reported performances.

In contrast to previous studies, we were able to choose different operating points for the AI system.

At our conservative operating point, the AI system remained sensitive for urgent or critical findings, only missing four urgent findings resulting in a calculated sensitivity of 99.2%, which was slightly lower than the sensitivity of 99.9% reported by Plesner et al. However, in our study, 53% of all normal chest radiographs were identified. This was considerably higher than the 28% in the study of Plesner et al. This may be explained by the fact that a different commercial AI system was used compared to the other studies. The reported sensitivity for significant abnormalities in the study by Keski et al was 99.8%. However, in this study, the reference standard was based on natural language processing on the original report, and only discrepancies between the radiological report and the AI system were reviewed by experts, which biased the performance of the AI system.

The optimal operating point may be different depending on the clinical situation. The default operating point led to the detection of 92% of all normal chest radiographs, potentially leading to a workload reduction of 36%. However, at this operating point also more radiographs were classified as normal that contained abnormalities according to our reference standard. This may not be clinically acceptable, and even with a conservative operating point, the AI system was not perfect. At the conservative operating point, the AI system still missed four urgent findings, and one of these misses was a CT-confirmed hilar mass. However, it is assumable that radiologists would have a comparable miss rate, as described in the literature [21, 25–28]. This is also reflected in the performance of the original report in the study by Plesner et al, in which the radiologist reached a sensitivity of 93.5% for ‘critical’ abnormalities. For broader acceptance of the use of autonomous AI systems, a conservative threshold should be chosen. To avoid any significant mistakes by the AI system an even more conservative operating point could be chosen. However, there is always a trade-off between the number of missed findings and the percentage of detected normal radiographs (i.e., the workload reduction). Another aspect to consider is that the AI system used in the study is not specifically designed to find normal radiographs, but rather to find the predefined ten distinct abnormalities seen on the chest radiograph. Therefore, chest radiographs that contain thoracic abnormalities outside the scope of the AI system may mistakenly be considered normal in our study. For instance, the AI system detects mediastinal widening, but only at the upper mediastinal level. This may be the reason why the hilar mass was missed.

Our study had a few limitations. First, the reference standard relies on expert opinion. We tried to avoid bias by including three experts in our reference standard. Moreover, to further optimize our reference standard, the experts had access to the original report which offered valuable clinical context for reviewing the radiographs. The reference standard might have been stronger when CT data was included. However, for the purpose of this study, we believe that using consecutive data outweighed the potential benefits of a CT-controlled set-up. Second, we did not include bedside or pediatric images in our analysis. On the other hand, the number of normal bedside chest radiographs is low, and bedside images are commonly used to monitor patients, and not to exclude disease. We included the bedside radiographs in the calculation of workload reduction, to get a fair estimate of potential time saving. Third, no external validation of the AI system has been performed. Although the AI system is commercially available, it was not designed or CE-marked to detect normal chest radiographs. External validation is useful to demonstrate the robustness of the AI system. Finally, the actual reduction in workload may be less than our reported 15%. The reading times for chest radiographs are short in general and even shorter for normal radiographs. Therefore, the more challenging and time-consuming cases still necessitate radiologist assessment. Moreover, the AI system is not yet approved for standalone reporting of (normal) chest radiographs, and therefore requires oversight by a human expert. Still, due to the high volume of chest radiographs, substantial time can be saved by expediting reporting or even removing the normal chest radiographs from the routine clinical workflow.

Overall, this study shows that a commercially available AI system can effectively identify normal chest radiographs and has the potential to reduce the workload for radiologists by eliminating a significant number of normal exams from human reporting. This has implications for improving the efficiency in radiology departments, especially in the context of increasing workload and the need to optimize healthcare budgets. However, further research and validation on larger datasets, as well as prospective studies are necessary to assess the generalizability and reliability of AI systems for chest radiography.

## Abbreviations

AI: Artificial Intelligence
AUC: Area under the curve
CT: Computed tomography
NPV: Negative predictive value
PPV: Positive predictive value
ROC: Receiver operating characteristics
